# I HAVE TO COPE WITH IT: THE VOICES OF OLDER AFRICAN IMMIGRANTS EXPERIENCING SOCIAL ISOLATION AND LONELINESS IN THE US

**DOI:** 10.1093/geroni/igac059.2620

**Published:** 2022-12-20

**Authors:** Dolapo Adeniji, Gifty Ashirifi

**Affiliations:** IUPUI, Avon, Indiana, United States; Indiana University, Indianapolis, Indiana, United States

## Abstract

Social isolation and loneliness have been recognized as significant challenges in the world of older adults. For older African immigrants living with their families in the US, researchers have captured factors such as language barriers, cultural differences, and limited access to transportation to contribute their feelings of social isolation and loneliness. However, little is known about how they cope with these challenges. As the population of older African immigrants continues to increase in the US, it is pertinent to expand knowledge about their experiences for the purposes of social work practice and policy development. Using a qualitative approach, this study recruited and conducted in-depth interviews with 11 participants aged 63 -79. Four themes emerged from the data through a thematic analysis approach which includes a) Positive Self-talk: “I have to cope with it”, b) Technology/Social media: "if I cannot interact physically outside, then, I go through the social media”/Watch TV”, c) Intergenerational social engagement beyond caregiving: "They [grandchildren] are my immediate constituency”, and d) Digging deep through faith. Although the result of this study shows that older Africans immigrants are finding strategies to cope with social isolation and loneliness further support is needed specially to strengthen their coping skills and enhance their social network with people outside of their families.

